# Nurses’ perspectives and experience in caring for patients undergoing hemodialysis at Benjamin Mkapa hospital in Dodoma, Tanzania: A qualitative study

**DOI:** 10.1371/journal.pone.0325501

**Published:** 2025-06-10

**Authors:** Stephania Mbunda, Neema E. Mawi, Masunga K. Iseselo

**Affiliations:** 1 Department of Clinical Nursing, Muhimbili University of Health and Allied Sciences, Dar es Salaam, Tanzania; 2 Department of Nursing Management, Muhimbili University of Health and Allied Sciences, Dar es Salaam, Tanzania; University of Luzon, PHILIPPINES

## Abstract

**Background:**

Caring for patients undergoing hemodialysis is a challenge for nurses. Poor quality of care in hemodialysis is reported to have a significant contribution to the high mortality rate among patients. Improving the health outcomes of the patient requires an in-depth exploration of the perspectives and views of nurses working in the hemodialysis unit. However, there is a paucity of information regarding the perspectives and experiences of nurses working in Hemodialysis units, particularly in resource-constrained countries like Tanzania. This study aimed to explore the perspectives and experiences of nurses caring for patients undergoing hemodialysis.

**Materials and Methods:**

An explorative qualitative study was conducted among nurses working in Hemodialysis units at Benjamin Mkapa Hospital in Dodoma, Tanzania. A purposive sampling method was used to select the participants. Ten in-depth interviews were conducted using a semi-structured interview guide and an audio recorder as the main data collection tools. A thematic analysis approach was used to analyze the data.

**Findings:**

Three themes and the associated subthemes emerged from this study. 1) Conducive environments for the provision of nursing care 2) Challenges affecting hemodialysis care, and 3) Strategies for improving hemodialysis care. A good nurse-patient relationship, financial incentives, and availability of equipment facilitate the provision of care among patients. Treatment costs, problems in adhering to dialysis prescriptions, and staff shortages were the challenges affecting hemodialysis care. On the other hand, increasing dialysis centres, training nurses, and free health insurance coverage were strategies for improving hemodialysis care.

**Conclusion:**

The findings highlighted several key aspects related to the perspectives and experiences of providing nursing care for patients undergoing hemodialysis. The conducive working environment, coupled with financial incentives, is an important factor in improving nurses’ care for patients undergoing hemodialysis. However, the high costs related to the utilization of hemodialysis services are the challenge to achieving better outcomes.

## Introduction

Chronic kidney disease (CKD) is a major public health concern that affects over 10% of people worldwide [1,2]. A growing number of people are losing their lives due to CKD, making it one of the major causes of death worldwide [2,3]. The burden of kidney diseases is higher in lower- and middle-income countries with limited health facilities for screening and treatment than in high-income countries [4]. In African countries, the overall prevalence of CKD ranges between 9·9–11·7% [5]. In Tanzania, few studies have been conducted to examine the magnitudes of CKD with marked variation in urban and rural areas [6–8]. For example, a study in Northern Tanzania reported that the community-based prevalence of CKD was 7.0%, with 15.2% in urban areas and 2% in rural settings [7]. The varied stages of sociodemographic and epidemiological health transitions in these areas may be partly responsible for the disparities in CKD prevalence and hazards [9]. Also, the risk of mortality and morbidity is higher for people with lower socioeconomic levels (SES) [10].

In Tanzania, the common treatment services for patients with end-stage kidney disease (ESKD) include kidney transplantation and dialysis. Hemodialysis is the most widely used and second-best treatment following kidney transplant that improves patients’ lifestyle and their quality of life for patients with renal failure [11]. Despite its benefits, hemodialysis treatment imposes several acute and chronic complications to patients including intestinal distress, infections, fluid overload, dialysis disequilibrium syndrome, hypokalemia, hypotension, and electrolyte abnormalities [12–14]. Moreover, evidence shows that patients undergoing hemodialysis experience higher levels of stress, with psychological stress being more prevalent compared to physical stress [15].

Nursing care provided in the hemodialysis units plays a critical role in improving patients’ health outcomes [16–18]. Nurses spend more time with patients compared to other healthcare professionals. Therefore, they are obliged to identify and tackle essential needs and care for hemodialysis patients [19]. Beyond providing technical support, nurses in this unit execute multiple roles, including the establishment of interpersonal and therapeutic relationships, managing physical symptoms, identifying and addressing mental health and educational needs of patients [20,21]. Besides that, they serve as a vital link between patients, other healthcare providers and patients’ relatives [22]. To provide optimal care, nurses require specialised training not only in technical support but also in comprehensive care of hemodialysis patients. However, literature shows a deficit of adequately trained nurses to provide quality dialysis services, particularly in Sub‐Saharan Africa (SSA) [23]. Furthermore, studies pointed out that factors such as nurses’ educational level, nursing skills, working experience, staffing, workload, job shift and the availability of mental and emotional support significantly influence the quality of care provided to hemodialysis patients [16,24–27].

Studies exploring nurses’ experiences of working with and caring for hemodialysis patients reveal both positive and negative aspects [16,24,28,29]. Positive experiences include improving patients’ quality of life; reducing physical problems and complications, increasing patients’ life expectancy and fostering interdependencies between patients and nurses [16,24]. On the other hand, reported negative experiences are physical and emotional exhaustion, depersonalization, burnout, neglect of family needs, and mental effects such as depression and anxiety [24]. These negative experiences not only impact nurses’ well-being but also affect the quality of care provided to patients.

In Tanzania, the number of health facilities offering hemodialysis services to patients with ESKD is rapidly increasing. The majority of nurses who constitute the largest workforce in hemodialysis units have not received specialised training. Instead, they rely on on-the-job training and short-term programs (2–3 months) that mainly focus on the technical aspects of operating dialysis machines [4]. Lack of adequate training may impact nurses’ confidence, competency, and the overall quality of nursing care. To our knowledge, this is the first study to explore nurses’ perspectives and experiences of working in hemodialysis units in Tanzania, despite the improvement of hemodialysis services in the country. Findings from this study will add to the body of knowledge on the experience of nurses and might help hospital administrators and other stakeholders to reinforce the positive aspects while addressing the challenges. Thus, this study explored the perspectives and experiences of nurses caring for patients undergoing hemodialysis treatment in Dodoma, Tanzania.

## Materials and methods

### Study design

An explorative qualitative study design was used to explore nurses’ perspectives and experiences in caring for patients undergoing hemodialysis. This study helps to identify positive experiences that need to be reinforced and changes that need to be addressed by responsible authorities hence improve patient care.

### Study setting

The study was conducted at the hemodialysis unit at Benjamin Mkapa Hospital (BMH) in Dodoma region, Tanzania. Dodoma is the capital city of Tanzania and is located in the central part of the country. BMH is a public tertiary hospital established in 2015 with a capacity of 400 beds that serves both in and outpatients from within and outside the country. It is the second hospital in the country to start offering kidney transplant services in Tanzania [4]. It receives patients from all over the country for hemodialysis treatment. According to unpublished data provided at the hospital, an average of 77 admissions of patients for hemodialysis treatment are recorded per day rendering a high burden of care among nurses.

### Participants, sampling and sample size

This study recruited registered nurses with a working experience of at least 1 year in a hemodialysis unit. Purposive sampling was used to select participants based on the information they had on caring for patients with ESRD. The first author identified and selected the potential study participants in consultation with the in-charge of the hemodialysis unit. For those who agreed to take part in the study, an individual appointment for the interview was arranged based on their convenience time. A total of 10 participants were recruited based on information saturation. This is the point when new data did not bring or contribute additional insights to the research questions during collection and data analysis [30]. Thus, in the context of this study, additional recruitment after the 10th participant did not yield any new insight.

### Data collection tool and procedures

The study utilised a semi-structured interview guide to gather information from the study participants. The guide consisted of questions constructed based on the literature review related to nephrology nursing scope and standards of practice [31]. Apart from questions that enquired about participants’ demographic characteristics, the interview guide consisted of the following questions: 1) What are your views on caring for patients undergoing hemodialysis? 2) What is your experience in managing patients’ symptoms that develop during hemodialysis? 3) What challenges have you encountered when providing nursing care to patients undergoing hemodialysis? 4) What are the coping strategies for reducing the challenges of hemodialysis treatment? (S1 File). These questions were followed by several probing questions to ensure that most of the information related to the experience of caring for hemodialysis patients was explored. Besides that, questions continue to be modified during the interview to unfold the emergent information related to the research objectives.

The data collection was conducted from the 15^th^ of May to the 10^th^ of June 2023 by the first author. The interview was carried out in quiet rooms on the hospital premises that were proposed by the study participants. This was important to enhance the neutral environment of the interview. The selected rooms were secluded and well-lit to minimize distraction and allow the exploitation of non-verbal cues from the study participants. The interviews were conducted in the Swahili language, which was common to all participants. All interviews were audio recorded with permission from the participants. Interviews lasted between 45 and 60 minutes.

### Data analysis

The data analysis started from the first interview and then continued throughout periods of data collection. Before analysis, the audio data were transcribed verbatim by the first author. The third author counterchecked the transcripts with the audio data to ensure consistency of information. Some omissions and typographic errors were observed, and appropriate corrective actions were made. The transcripts were then read and re-read to familiarize with the data. The first author translated the transcripts into the English language as per the guidelines [32] and was validated by the third author, who was more experienced in qualitative research before coding.

A thematic analysis method was used to analyze data as stipulated by Naeem et al. [33]. All authors were involved in the coding of the transcripts. Initial codes were generated independently using various approaches. The first author coded the data manually, while the second and third authors used NVivo 12 to organize and code the data. Every transcript was read several times to identify the concepts and ideas that could match with the appropriate codes generated from the literature review (deductive codes). After generating initial codes, the authors shared the codes to agree on the common codes. The sharing of codes that were produced independently enhanced the reliability of the codes. We then collated and combined the codes to generate meaningful themes. The authors reviewed the generated themes by checking if they correlate with the coded extract and the entire dataset; hence otherwise gathered a thematic map of analysis to find out whether they are meaningful and answer the research questions. When the initial themes were confirmed to be in line with the objective, naming and refining the themes were done to ensure that each theme tells the stories about the context from the participants’ perspectives. Based on similarities and differences identified, codes were re-organized and combined to form themes and sub-themes that were supported by participants’ quotes.

### Trustworthiness

The credibility, dependability, conformability, and transferability of the data were evaluated to determine their rigour and reliability using the Ahmed criteria [34]. To ensure credibility, the researchers gathered and examined the field data over a prolonged period. To increase reliability, peer debriefing and ongoing data comparison were used. To obtain external validation, codes were shared among all the authors to come up with common final codes that were used to form the themes. Sampling mode, question development, the method of coding, and theme formation and change were documented to provide appropriate conformability.

### Ethical approval

Ethical approval was obtained from the Research and Ethics Committee of Muhimbili University of Health and Allied Sciences (MUHAS) with Ref. No: DA.282/298/01.C/1607. Permission to collect data was obtained from BMH management. All potential participants were informed about the aim of the study, methodology, confidentiality, and potential risks and benefits. Additionally, they were informed that participation was voluntary and there were no consequences for deciding not to participate. Participants who agreed to participate in the study were asked for written informed consent before starting the interview. Consented participants were informed of the freedom to skip any question and to stop the interview at any time. To protect the anonymity of the study participants, names and other identifying information were not collected.

### Findings

#### Socio-demographic characteristics of participants.

A total of ten participants participated in this study. Participants age ranged from 23 to 39 years. More than half of the participants (6) were male and the majority (8) were working as Assistant Nurse Officers (diploma level). The working experience in the hemodialysis unit varied from 1 to 5 years (Table 1).

**Table 1:** Social demographic characteristics of participants.

Participant No	Age (years)	Working experience in a hemodialysis unit (Years)
1	27	1 year
2	23	1 year
3	26	3 years
4	32	5 years
5	36	3 years
6	26	3 years
7	33	3 years
8	37	1 year
9	39	2 years
10	32	5 years

#### Themes and sub-themes.

In this study, three themes and nine sub-themes emerged. The first theme described a conducive working environment. In this theme, good nurse-patient relationships, the provision of incentives to nurses, and the availability of equipment are the three subthemes that emerged. The second theme describes challenges affecting dialysis care with three sub-themes, namely treatment cost, problems in adhering to hemodialysis, and staff shortage. The last theme describes the strategies for improving hemodialysis care, which has also constituted increasing hemodialysis centres, on-the-job training of nurses, and free health insurance coverage (Table 2).

**Table 2:** Themes and sub-themes.

Themes	Sub-themes
Conducive working environment	1. Good nurse-patient relationship 2. Financial incentives to nurses 3. Availability of equipment
Challenges for Hemodialysis Care	1. Cost of treatment 2. Inconsistency in hemodialysis prescription 3. Staff shortage
Suggested Strategies for Improving Hemodialysis Care	1. On-the-job training of nurses 2. Increasing hemodialysis centers

### Theme 1: Conducive working environment

#### Good nurse-patient relationship.

Participants expressed that the nurse-patient relationship was an important aspect of providing good nursing care to the patients. They described that a good relationship with patients undergoing hemodialysis at their dialysis centres was linked to the patients’ outcomes. A good nurse-patient relationship creates a positive atmosphere for patients to express their needs and concerns to nurses freely. As was reported by participants:

*“… We have good cooperation and a relationship with the patients. This is because the patients are the same, we see them regularly, three times a week. Therefore, we are very used to them as a family, so to be honest, we live well without problems”* (P1, 1 year experience)*.*

On the other hand, participants described that patients often prefer to be attended by the same nurses whom they are used to throughout hemodialysis treatment. They expressed that patients tend to be more comfortable and have faith in services provided by nurses who had served them before or ever interacted. This enhanced trust and cooperation between the two parts as evidenced by one of the participants below:

“*Patients who are used to dialysis prefer to be cared for by the same nurse. If for example, a new nurse was brought in; may say mmm! I do not have faith in him, I do not think he can serve me well. But if they are used to them! That does not exist*” (P5, 3 year experience).

The participants with a positive perception of the working environment in hemodialysis expressed a deep sense of fulfilment. They described the environment as encouraging because it was a space where compassion and expertise intersect. They believed that their work made a significant difference in the lives of their patients daily. The friendly relationship among the healthcare team and the shared sense of purpose of patients created an uplifting atmosphere that keeps them motivated to provide the best care possible. Participants expressed a feeling of a strong connection to the work and joyous environment that gives them satisfaction.

#### Financial incentives to nurses.

Participants described that the provision of financial incentives improved the motivation of nurses. They verbalized those incentives such as extra duty allowance increased their morale of caring and hence improved the patient outcome in the dialysis unit as described by the participants below:

*“Giving incentives is a good thing and we just enjoy it all, we are transparent to each other how much motivation we have received, who has not received, then we send the information to our leader who is the in charge” (*P1, 1-year experience).

Moreover, participants reported that although they get financial incentives, it was given according to the number of National Health Insurance Fund (NHIF) patients they served. Therefore, the more NHIF patients they served, the more they got financial incentives and vice versa. This motivated the participants to work hard knowing that they would receive financial gain after completing the assigned tasks. This had improved their optimism towards working in the dialysis unit reported:

*“In terms of incentives, we are given according to the patients you served because we depend on Tanzania NHIF or working overtime is when you are paid extra duty allowance according to your education level. But if you serve many NHIF patients, you get good motivation, if the patients decrease, your allowance also decreases because your work is reduced* “(P4, 5 years experience)

#### Availability of equipment.

Participants stated that the availability of equipment was essential for the provision of nursing care to patients undergoing hemodialysis. They verbalized that when equipment is available at the unit nurses carry out procedures to a required standard that yields better patient outcomes than when equipment is limited. They expressed that a good and constant supply of essential equipment contributed to the provision of effective nursing care to patients undergoing hemodialysis. They added that the hospital had equipment that was available all the time as narrated by one of the participants:

*“In terms of equipment, we are grateful; are available, even if sometimes there are delays in ordering or getting the supplies from the Medical Stores Department (MSD) but not to the extent that we run out of stock to affect the provision of care. But most of the time, we have access to the necessary equipment for providing the service.”* (P8, 1 year experience)

### Theme 2: challenges for hemodialysis care

#### Cost of treatment.

Participants described that undergoing three sessions of hemodialysis per week was very expensive for many patients to afford, especially those without health insurance from poor families. The participants reported not affording the cost associated with hemodialysis despite the effort by the Tanzanian government to reduce the cost to the minimum of Tsh 300,000 (120 USD) per session. The high cost of care described to be compounded by delays reaching the dialysis centre due to long distances and unreliable transport as evidenced by the following participants’ statements:

*“… For our customers who pay in cash, we have now seen that the government has made efforts to reduce the cost of dialysis which has slightly decreased, but still for an ordinary Tanzanian, especially someone in a lower income status paying for this treatment is very expensive.”* (P9, 2 years experience)

Health care coverage ensures access to health care services including hemodialysis services. Participants explained that when patients undergoing hemodialysis are covered with free healthcare insurance will attend dialysis sessions without missing because they would not be required to pay the associated treatment cost except for their transport to the health facility as commented by the participants:

*“For a patient without NHIF coverage, it is a challenge for them to afford three sessions of dialysis per week. A patient needs to pay 300,000 Tanzanian shillings per session. Many patients cannot afford it. If the government provides free health insurance coverage to patients will increase access to hemodialysis care” (*P10, 5 years experience)

Furthermore, participants were concerned with the coverage of health insurance schemes used by the patients undergoing hemodialysis. Participants reported that some insurance schemes do not cover dialysis services making patients pay out of pocket. As reported:

*“Some health insurance schemes do not include expensive health services like dialysis. This is a challenge because patients undergoing hemodialysis most of the time are financially comprised to afford dialysis”* (P9, 2 years experience)

#### Inconsistency in hemodialysis prescription.

Participants accounted that non-adherence to hemodialysis prescription was another challenge that affected the progress and treatment outcomes. It was pointed out that sometimes nurses do not adhere to dialysis prescriptions for various reasons including improper documentation of patients’ particulars. This could be caused by a lack of experience and knowledge on the proper recording of the information in the case note as elaborated by the participant below:

*“I have heard of dialysis prescription, but based on my experience, it is not something that is normally adhered to practice. When a patient comes, we already know how we are going to treat them, and if it happens to be done it is not done regularly”* (P8, 1-year experience)

Additionally, nurses reported that most of the time patients arrive when doctors are not around. Therefore, nurses continue to provide care to patients because they already know how to manage these patients. Sometimes this is performed below the standard as in the description below:

*“Hemodialysis prescription is not practiced properly because we start to attend to patients when the doctor who is supposed to prescribe is not there, but we also know that the body weight that he left within the last session and the body weight that he has today may be erroneously recorded.* (P6, 3 years experience)

#### Staff shortage.

Participants described that staff shortage was another significant challenge that affected the entire process of provision of care to patients undergoing hemodialysis. Participants stated that the centre had 13 nurses in total but normally 3 nurses were on off-duty every day. Therefore, only 10 nurses were serving 77 patients per day. This further shows that one nurse in the dialysis unit could take care of five to six patients per day which is above the recommended guideline. They added that, with such a shortage, it would be difficult for nurses to provide quality care to patients undergoing hemodialysis, as expressed below:

*“The challenge we have is the shortage of staff, we may attend to about thirty patients in a shift and nurses working in the morning shift may be four or five staff. Therefore, you cannot manage to sit with each patient to know their challenges well. When they arrive, we take them straight to the machine. We do not do a thorough assessment because of the limited time of the number of patients waiting for the same services. So, we just check the blood pressure and continue*” (P1, 1 year experience)

### Theme 3: suggested strategies for improving hemodialysis care

#### Increasing hemodialysis centres.

Participants expressed the challenge experienced by patients undergoing hemodialysis to reach the dialysis centre due to distance. They said that patients for hemodialysis had to travel a long distance to get hemodialysis. They added that the facilities that provide hemodialysis were not proportional to the number of patients who needed the service. To improve hemodialysis care and reach many patients, participants suggested establishing several dialysis centres in the country which could be a reasonable solution for reducing the treatment costs of patients. They also noted that weak patients could hardly reach the hospital to receive the required services. Participants reported that some patients could not comply with the dialysis regimen because of the long queues waiting for the service at the dialysis centre, as evidenced by the participant below:

*“It is a problem because sometimes when patients come for dialysis, they find a very long queue. Sometimes they spend half of the day waiting for dialysis, but when more centres are available, they will not have to wait for long”* (P2, 1-year experience)

#### On-job training of nurses.

Participants also asserted that on-the-job training of nurses to ensure the provision of quality nursing care is significant. They reported that nurses need training to ensure quality nursing care to patients undergoing hemodialysis. Moreover, participants expressed that due to the rapidly changing healthcare environment, nurses need to use their knowledge to curb the changing technology as reported by one of the participants below:

*“True mastery in nursing lies not only in the skills we possess but in the unwavering commitment to continuous learning whenever the opportunity comes we keep learning. I recognize that knowledge is a powerful tool that empowers us to deliver exceptional care, navigate the ever-changing healthcare system, and truly transform lives with our compassion and expertise* “(P7, 3 years experience)

Other participants stated that a lack of continuous education might jeopardize the patients’ safety since the knowledge they had was outdated. Training of nurses enhances knowledge and skills in managing patients. Participants also reported a lack of regular refresher on-the-job training to improve their knowledge and skills to better the quality of services. As reported by the participant:

*“We get the training very rarely. The training is mostly organized by our in charge, so that we get it otherwise. Short of that, we do not get training”* (P6, 3 years experience)

## Discussion

This study explored the experience of nurses in caring for patients undergoing hemodialysis. A conducive environment for the provision of nursing care, challenges affecting patient care, and strategies for improving hemodialysis care are the main themes identified in the current study. Good nurse-patient relationships, the provision of incentives to nurses, and the availability of equipment are the subthemes revealed in the first theme. Cost of treatment, problems in adhering to hemodialysis prescriptions and shortage of staff were the challenges affecting dialysis care. Increasing dialysis centres and tailored on-the-job training of nurses are the suggested strategies for improving hemodialysis care in this study.

The good nurse-patient relationship as reported in this study indicates that nurses create and maintain good therapeutic interactions with patients as a driver for effective communication skills. It also shows that study participants have good therapeutic interactions; an important component of nursing care [35,36]. Furthermore, the good nurse-patient relationship revealed in our study indicates that the relationship can influence the sense of security, help in coping with difficult moments, and increase the quality of medical care [28,29]. This promotes therapeutic alliance in healthcare facilities. The good nurse-patient interaction that facilitates effective quality nursing care has been reported in other settings Italy [37], Iran [38] and Poland [39] whereby when the nurse-patient relationship is maintained, increases the efficiency and effectiveness of care to patients. Molina-mula and Colleague [40] recommended that the nurse-patient relationship should not pursue the change in values and customs of the patient, but position the professional as a witness of the experience of the health and illness process in the patient and family. This is an important aspect that needs to be maintained in all nursing care environments, particularly in the hemodialysis unit.

The provision of incentives, especially financial incentives to nurses working in the hemodialysis centre, improves their motivation and hence facilitates a positive and conducive environment for the provision of nursing care. This connotes that incentives improve the motivation of nurses, hence the provision of high-quality dialysis care. Evidence has shown that when nurses are motivated, they are self-directed, requiring minimal supervision [41,42]. However, the issues of financial incentives and quality of care are still controversial. Abduljawad and Al-Saaf [43] in their study reported a mixed effect on performance after financial incentives. Also, a systematic review in low and middle-income countries(LMICs) found limited evidence of successful interventions to motivate health workers in LMICs [44] and recommended robust studies that use validated and culturally appropriate tools to assess healthcare providers’ motivation about financial incentives.

The current finding is supported by a study reported by Atalla et al. in Egypt showed that incentives motivate nurses, and there is a positive correlation between nurses’ motivation and patients’ satisfaction [45]. However, contrary to our findings, other studies have revealed that health workers are strongly motivated by non-financial incentives [46,47]. This is an interesting area that need further exploration, particularly for nurses working in the hemodialysis unit.

The availability of essential equipment for the provision of care to patients undergoing hemodialysis is an important finding reported in this study. This finding indicates that the hospital management ensures the hemodialysis unit is supplied with the necessary equipment to smoothen the provision of dialysis care to patients. Contrasting findings were identified in India that revealed a significant lack of resources in dialysis centres, making the provision of highly technical and expensive care [48]. Also, the current study is opposed by the findings in Nigeria which showed a multiple problems in operating the hemodialysis machines including frequent break down, absence of adequate maintenance technical support and spare parts, and power outages [49].

It is revealed in the current study that staff shortage is a challenge to dialysis care. This can be described by a limited number of nurses trained in nephrology and working in hemodialysis units [4]. This means that there is a high number of patients who need care compared to the number of nurses providing care at the hemodialysis unit. Our finding shows a far less than the recommended nurse-to-patient ratio of 1:10 for caring for patients with ESRD [50]. The association between nurse staffing and adverse patient events in this study is not surprising. Numerous studies in hospitals have consistently demonstrated a significant relationship between low nurse staffing levels and adverse patient outcomes, including higher mortality rates and lower patient satisfaction levels [51,52]. A high nurse-patient ratio affects the provision of effective, quality nursing care. The finding is in line with that reported in the USA, which revealed that for over a decade, the country had been experiencing a shortage of nephrology nurses, including those with dialysis expertise [53]. The staff shortage can also be described by the fact that hemodialysis is a new treatment technology in Tanzania, in which most nurses have not been trained(4). In addition, a meta-analysis revealed that a nurse-led disease management improved the quality of life in terms of symptoms, sleep, staff encouragement, pain, general health perception, energy/fatigue, and overall health [18]. Thus, investment in Human resources for health is an important step towards the improvement of hemodialysis care.

The inconsistency in the hemodialysis prescription as revealed in our study indicates that nurses need awareness of the global and national guidelines [54,55]. It is well documented that non-adherence to hemodialysis attendance and prescription is associated with increased mortality [56]. Adherence to prescription can lower mortality and enhance wellness, as outlined in the standard documents.

This study revealed that the cost of dialysis affects dialysis care. Due to the expensive nature of hemodialysis services, most patients, particularly those with low income, can to afford it. The finding in this study is in line with that reported by Mushi et al. [57], in Dar es Salaam, Tanzania, in which the average unit cost per hemodialysis was 176 US$. The fact that hemodialysis and other nephrology services are covered by the National Health Insurance Funding Scheme (NHIF) suggests that other Tanzanians who are not members must pay for them out of pocket [4,58]. Our study implies that the costs of hemodialysis care to patients are high, such that the government should provide public subsidies for dialysis and expand social health insurance coverage to reduce the costs. Lack of fare for transport and long distance to the hemodialysis centre, as reported in our study, further exacerbate the burden of treatment cost among the patients. This might have been caused by a shortage of hemodialysis units in the vicinity of the users.

The suggestion that more hemodialysis centres should be established is an important step to the improvement of the services in Tanzania, as reported in other studies [56,59]. Establishing hemodialysis centres near the patients may not only reduce the cost of treatment but also improve the well-being of the patient. In 2019, the number of dialysis units in Tanzania was 29 which were unevenly distributed in 7 regions [4]. This number is not enough to cater all the increasing number of patients with ESRD in the country. Inadequate hemodialysis units lead to delays in seeking care, which has detrimental consequences, including more comorbidities, poorer clinical and biological profiles and increased mortality [60,61]. On-the-job training is also an important factor for improving the skills of nurses who provide hemodialysis care and should be considered in health facilities which provide hemodialysis care.

### Limitation

This study was conducted at Benjamin Mkapa Hospital, a zonal and tertiary hospital in Central Tanzania. The scope and intensity of nursing care in tertiary hospitals and normal dialysis centres may be different be different hence limiting the applicability for nurses in the primary care. However, these findings provide insights into similar settings in Tanzania. Also, participants had a tight work schedule that might have affected their responses during the interview. In such situation, the interviews were scheduled and conducted based on the participants’ convenient time. This provided ample time for participants to be well relaxed during the interview. In addition, the use an interview guide in Kiswahili might have lost the original meaning of the participants during the translation process to English. The effect of the loss had been minimized by the researcher the use of bilingual (Swahili and English) experts who compared and validated the translated data process.

## Conclusion

The findings highlighted several key aspects related to the perspectives and experiences of providing nursing care for patients undergoing hemodialysis. The conducive working environment, coupled with financial incentives, is an important factor in improving nurses’ care for patients undergoing hemodialysis. Increasing the hemodialysis centres may improve the patient’s access to hemodialysis services. However, the high costs related to the utilisation of hemodialysis services are the challenge to achieving better outcomes. Future research should be conducted on the patients’ perspectives on the hurdles of undergoing hemodialysis in similar settings.

## Supporting information

S1 File Interview Guide. (PDF)
