# Supplementary material for: Nurses’ perspectives and experience in caring for patients undergoing hemodialysis at Benjamin Mkapa hospital in Dodoma, Tanzania: A qualitative study
Source: PLoS One. 2025 Jun 10;20(6):e0325501. doi: 10.1371/journal.pone.0325501 (PMC12151407; doi:10.1371/journal.pone.0325501)
Supplement: S1 File — (PDF) [file pone.0325501.s001.pdf]

Supporting Information: Interview Guide

<u>Interview Guide for Nurses Working with Patients Undergoing Hemodialysis Treatment</u>

The *guide* contains the questions that will be asked during the *interviews* with nurses working with patients undergoing hemodialysis treatment.

1. What is your experience with the management of patients undergoing hemodialysis care?
2. What is your experience with the symptoms that patients typically experience in hemodialysis? What efforts do you consider to assume responsibility for your patients' overall health?
3. What are the challenges you encounter when assessing patients for hemodialysis? Situation or environment? Privacy? Assessment devices? Patients' pathway?
4. What are the challenges for caring patient undergoing Hemodialysis treatment? Can you give examples when contributing your responses? organizational? relationship with patients? guideline? motivation? communication?
5. What are your opinions on reducing the existing challenges for Hemodialysis treatment? Be open, and can you give examples when contributing your responses?
6. What do you think your organisation lacks to support you in maintaining the nursing standard of care for patients undergoing hemodialysis? Probe, supervision? Incentives or motivation? Working conditions? workload? material resources? Priority? Regular training support?
7. Do you have any questions or suggestions?

Thank you for your participation. This marks the end of our interview.
